# Indole-3-Propionic Acid, a Gut Microbiota-Derived Tryptophan Metabolite, Promotes Endothelial Dysfunction Impairing Purinergic-Induced Nitric Oxide Release in Endothelial Cells

**DOI:** 10.3390/ijms25063389

**Published:** 2024-03-16

**Authors:** Federica Geddo, Susanna Antoniotti, Maria Pia Gallo, Giulia Querio

**Affiliations:** Department of Life Sciences and Systems Biology, University of Turin, Via Accademia Albertina 13, 10123 Turin, Italy; federica.geddo@unito.it (F.G.); susanna.antoniotti@unito.it (S.A.); giulia.querio@unito.it (G.Q.)

**Keywords:** cardiometabolic health, endothelial dysfunction, gut microbiota, indole-3-propionic acid, nitric oxide

## Abstract

Different gut microbiota-derived metabolites influence cardiovascular function, and, among all, the role of indole-3-propionic acid (IPA), from tryptophan metabolism, shows controversial effects. The aim of this study was to evaluate its role in endothelial dysfunction. IPA effects were studied on bovine aortic endothelial cells (BAE-1). First, IPA cytotoxicity was evaluated by an MTS assay. Then, the levels of intracellular reactive oxygen species (ROS) were evaluated by a microplate reader or fluorescence microscopy with the CellROX^®^ Green probe, and nitric oxide (NO) production was studied by fluorescence microscopy with the DAR4M-AM probe after acute or chronic treatment. Finally, immunoblotting analysis for endothelial nitric oxide synthase (eNOS) phosphorylation (p-eNOS) was performed. In BAE-1, IPA was not cytotoxic, except for the highest concentration (5 mM) after 48 h of treatment, and it showed neither oxidant nor antioxidant activity. However, the physiological concentration of IPA (1 μM) significantly reduced NO released by adenosine triphosphate (ATP)-stimulated BAE-1. These last data were confirmed by Western blot analysis, where IPA induced a significant reduction in p-eNOS in purinergic-stimulated BAE-1. Given these data, we can speculate that IPA negatively affects the physiological control of vascular tone by impairing the endothelial NO release induced by purinergic stimulation. These results represent a starting point for understanding the mechanisms underlying the relationship between gut microbiota metabolites and cardiometabolic health.

## 1. Introduction

Approximately ten trillion microbial cells colonize the gastrointestinal tract, especially the gut, where they carry out various physiological functions impacting the host’s health, such as the fermentation of not-digested dietary fibers, vitamin synthesis, regulation of the immune system, and the maintenance of the intestinal epithelial mucosal barrier [1]. Gut dysbiosis, the alteration in microbiome composition, is correlated with the pathogenesis of, among others, intestinal disorders, type 1 diabetes, and obesity [2]. In particular, there is growing interest in studying the relationship between gut microbiota and cardiovascular diseases [3]. Indeed, the gut microbiota is widely considered to be the body’s largest endocrine organ due to its ability to generate biologically active metabolites that can be absorbed into circulation and that positively or negatively affect cardiovascular function, showing up the so-called gut–heart axis [4]. For example, short-chain fatty acids (SFCAs), produced in large quantities by the gut microbiota through the anabolic fermentation of dietary fibers, seem to improve cardiac function and confer post-infarction cardiac protection [5,6,7]. On the other hand, the microbiota-dependent metabolism of food-derived choline, L-carnitine, and ergothioneine produces trimethylamine (TMA), which enters portal circulation and is oxidized by the hepatic flavin monooxygenases to trimethylamine N-oxide (TMAO), which is associated with adverse cardiovascular events, including endothelial dysfunction, atherosclerosis, and acute heart failure. Besides these, emerging evidence suggests that gut microbiota-derived tryptophan metabolites impact the host’s homeostasis, exerting physiological and pathological functions [8,9,10,11]. Tryptophan (Trp) is an essential amino acid that cannot be synthesized in the human body and must be introduced through diet [12]. Trp can be metabolized through the kynurenine pathway by the enzyme indoleamine-2,3-dioxygenase-1 (IDO1) in immune cells and intestinal epithelial cells, or the serotonergic pathway by the enzyme Trp hydroxylase (TpH) in the gut (TpH1) or in the brain (TpH2), which converts a small part of Trp into serotonin (5-hydroxytryptamine), which is further transformed into melatonin [12,13]. In addition to the endogenous metabolism, a significant portion of diet-derived Trp is directly metabolized by intestinal microorganisms, principally through Trp aminotransferase (TAA), into several biologically active groups of indoles and their derivatives, including indole-3-propionic acid (IPA) [12,13]. Over the past years, several studies have highlighted the antioxidant, anti-inflammatory, anti-cancer, and neuroprotective effects of IPA [14,15,16,17]. Most of these studies concur in identifying the aryl hydrocarbon receptor (AhR) [18,19] and the pregnane X receptor (PxR) [20,21] as the main targets of the biological activities of IPA. Although the protective effects are well known, the cardiovascular effects of IPA are still debated. Indeed, on the one hand, there is some evidence that IPA deficiency is negatively correlated with atherosclerosis [22,23]. Still, on the other hand, other results suggest that IPA increases blood pressure in rats [24], alters cardiac function in cardiomyocytes [25], and correlates with vascular inflammation and atherosclerosis [26].

Since IPA’s role in vascular health is still unclear, this study aimed to evaluate its effect on endothelial dysfunction (ED), a hallmark in the development of atherosclerosis and other cardiovascular diseases [27,28]. Given that endothelium plays a key role in modulating vascular tone through the production and release of different relaxing factors, including nitric oxide (NO), the loss of these physiological functions results in the inability to control vascular homeostasis and the impairment of vascular smooth muscle relaxation [28,29]. In particular, the effect of IPA was studied on bovine aortic endothelial cells (BAE-1) both with acute (30 min) and chronic (24 h) treatment, taking into account the key elements involved in vascular health: reactive oxygen species (ROS) intracellular level and NO production. Moreover, since the reduction in NO release is due to the impaired activity of endothelial nitric oxide synthase (eNOS), we evaluated the phosphorylation level of the enzyme at Ser 1179 (1177), the most important regulatory phosphorylation site that promotes eNOS activation [30]. 

## 2. Results

### 2.1. IPA Does Not Affect BAE-1 Cells Viability

**Figure 1:**
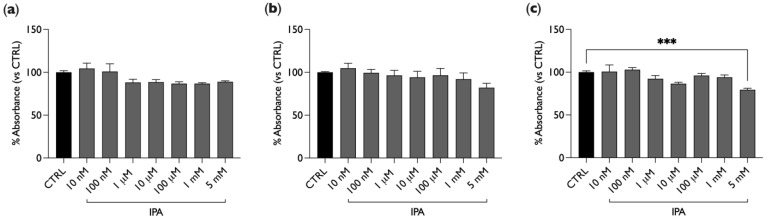
IPA does not significantly affect cellular viability. BAE-1 cells were treated with different concentrations of IPA for 4 h (**a**), 24 h (**b**), or 48 h (**c**), and then an MTS assay was performed. Data, expressed as a percentage referring to the control condition (untreated cells), are represented as mean ± SEM, *** *p* < 0.001. Values of the n = 3 independent experiments were as follows: (**a**) CTRL: 100.00 ± 1.98; IPA 10 nM: 104.40 ± 6.32; IPA 100 nM: 100.98 ± 8.95; IPA 1 μM: 88.20 ± 3.80; IPA 10 μM: 88.60 ± 2.83; IPA 100 μM: 86.80 ± 2.15; IPA 1 mM: 86.86 ± 1.11; IPA 5 mM: 89.00 ± 1.03; (**b**) CTRL: 100.00 ± 0.78; IPA 10 nM: 104.79 ± 5.79; IPA 100 nM: 99.44 ± 4.06; IPA 1 μM: 96.44 ± 5.87; IPA 10 μM: 94.24 ± 6.97; IPA 100 μM: 96.55 ± 7.99; IPA 1 mM: 92.10 ± 7.21; IPA 5 mM: 82.12 ± 5.02; (**c**) CTRL: 100.00 ± 1.54; IPA: 10 nM 100.69 ± 7.70; IPA 100 nM: 102.88 ± 2.47; IPA 1 μM: 92.37 ± 3.66; IPA 10 μM: 86.40 ± 1.63; IPA 100 μM: 96.03 ± 2.50; IPA 1 mM: 94.06 ± 2.62; IPA 5 mM: 79.40 ± 1.78. IPA = indole-3-propionic acid.

In order to study the effect of IPA on cellular viability, BAE-1 cells were plated in 96-well plates and treated with different concentrations of IPA, ranging from nM to mM (10 nM, 100 nM, 1 µM, 10 µM, 100 µM, 1 mM, 5 mM). Viability was evaluated by means of the CellTiter 96^®^AQueous One Solution Colorimetric Assay; staining with MTS was performed after 4, 24, or 48 h of treatment with IPA. As shown in Figure 1, IPA did not significantly affect cellular viability at all three exposure times, even at the highest doses, except for 5 mM and 48 h of treatment (20% decrease, Figure 1c). 

### 2.2. IPA Does Not Show Oxidant or Antioxidant Activity

The role of IPA in reactive oxygen species (ROS) balance was evaluated in BAE-1 cells using two techniques, a fluorescence microplate reader and fluorescence microscopy.

For experiments where ROS production was analyzed at the microplate reader, BAE-1 cells in 96-well plates were treated with the stressor menadione (MEN) as a positive control (20 µM for 1 h) or with IPA (100 nM, 1 µM, and 100 µM) for 30 min or 24 h, alone or in combination with MEN. Then, the cells were labeled with a 5 µM CellROX^®^ Green probe, and the emitted fluorescence was acquired by a microplate reader.

**Figure 2:**
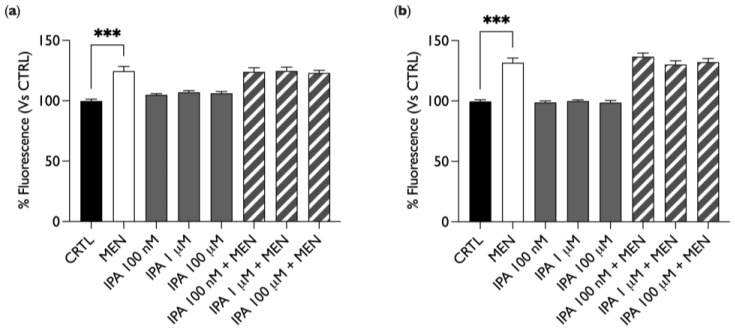
IPA does not affect ROS production in BAE-1 cells. Bar graphs summarize the effect on ROS production after treatment with 20 µM of MEN for 1 h or IPA (100 nM, 1 µM, and 100 µM) for 30 min (**a**) or 24 h (**b**) alone or in combination with MEN; BAE-1 cells were loaded with CellROX^®^ Green probe, for which fluorescence was recorded by a microplate reader. Data expressed in percentages which referred to the control condition (untreated cells) are represented as mean ± SEM, *** *p* < 0.001. Values of the n = 3 independent experiments were as follows: (**a**) CTRL: 99.83 ± 1.53; MEN: 124.51 ± 3.88; IPA 100 nM: 104.93 ± 1.03; IPA 1 μM: 107.08 ± 1.34; IPA 100 μM: 106.24 ± 1.41; IPA 100 nM + MEN: 123.92 ± 3.40; IPA 1 μM + MEN: 124.69 ± 3.15; IPA 100 μM + MEN: 123.04 ± 2.20; (**b**) CTRL: 99.74 ± 1.36; MEN: 131.69 ± 3.93; IPA 100 nM: 98.79 ± 1.22; IPA 1 μM: 99.93 ± 1.02; IPA 100 μM: 98.73 ± 1.72; IPA 100 nM + MEN: 136.71 ± 2.96; IPA 1 μM + MEN: 130.16 ± 3.22; IPA 100 μM + MEN: 132.17 ± 3.04. IPA = indole-3-propionic acid, MEN = menadion.

As shown in Figure 2, IPA did not increase the production of ROS, unlike MEN, which, as expected, induced a fluorescence increase. Moreover, in cells treated simultaneously with MEN and IPA, there was no reduction in fluorescence intensity as compared to MEN treatment only. This result indicates that IPA does not induce by itself the production of ROS but, at the same time, it does not show any antioxidant activity either since it does not counteract the effect of the oxidative stressor. Similar results were obtained with both acute (30 min, Figure 2a) and chronic (24 h, Figure 2b) IPA stimulation.

**Figure 3:**
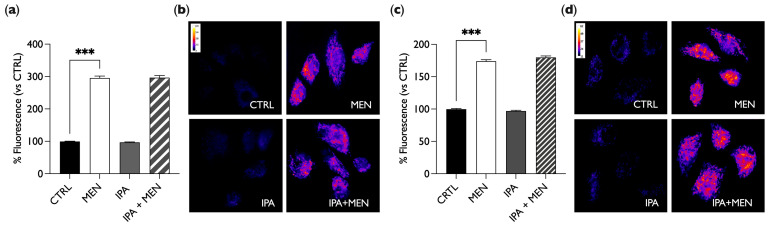
IPA does not affect ROS production in BAE-1 cells. (**a**,**c**) Bar graph summarizing the effect on ROS production after treatment with 20 µM of MEN for 1 h or 1 µM of IPA for 30 min (**a**) or 24 h (**c**) alone or in combination with MEN in comparison to the control (untreated cells). Data in percentages referring to the control condition are represented as mean ± SEM, *** *p* < 0.001. Values of the n = 3 independent experiments were as follows: (**a**) CTRL: 99.18 ± 1.02, n cells = 157; MEN: 294.41 ± 6.29, n cells = 173; IPA: 96.79 ± 1.20, n cells = 166; MEN + IPA: 295.03 ± 6.85, n cells = 172; (**c**) CTRL: 100.00 ± 1.14, n cells = 171; MEN: 173.98 ± 2.33, n cells = 167; IPA: 97.14 ± 1.10, n cells = 174; MEN + IPA: 179.77 ± 2.24, n cells = 153. (**b**,**d**) Representative fluorescent images (50×) of CellROX^®^ Green Reagent-labeled cells, treated for 30 min (**b**) or 24 h (**d**). Images are presented in pseudocolor (LUT = fire) to better show the fluorescence intensity variations (range 7–69 (**b**), 11–62 (**d**)). IPA = indole-3-propionic acid, MEN = menadion.

To further evaluate any effect of IPA on ROS levels, we performed live cell imaging with fluorescence microscopy experiments by using the CellROX^®^ Green probe and the stressor menadione (MEN) as a positive control. For these and subsequent experiments, a 1 µM IPA concentration was used, as this approaches the physiological concentration found in human serum [31]. BAE-1 cells grown on glass-bottom dishes were treated with 20 µM of MEN for 1 h or with 1 µM of IPA for 30 min or 24 h, alone or in combination with MEN, and they were then labeled with CellROX^®^ Green Reagent and analyzed by fluorescence microscopy. As shown in Figure 3, for both treatment times, IPA did not induce ROS production, unlike MEN, which conversely induced a significant fluorescence intensity increase compared to the control (cells maintained in DMEM + 10% FCS). Moreover, in cells simultaneously treated with MEN and IPA, there was no reduction in fluorescence intensity compared to the only MEN treatment, pointing out that IPA did not reduce the ROS increase induced by MEN.

These results confirm that IPA has a neutral role with respect to oxidative stress as it does not affect ROS, neither as an inducer nor as a suppressant of their intracellular production. 

### 2.3. IPA Reduces NO Release Induced by ATP

**Figure 4:**
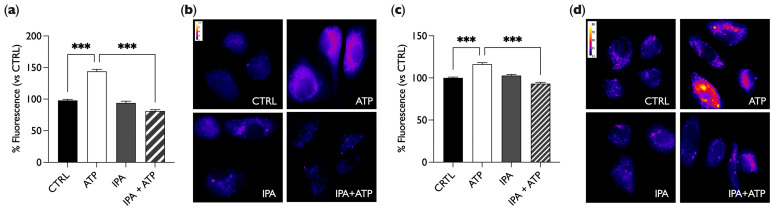
IPA reduces ATP-induced NO release. (**a**,**c**) Bar graphs summarize the effect on NO release after the stimulation with 100 µM of ATP and 1 µM of IPA, alone or together, compared to the control after 30 min (**a**) or 24 h (**c**) of IPA treatment. Data in percentages referring to the control condition are represented as mean ± SEM, *** *p* < 0.001. Values of the n = 3 independent experiments were as follows: (**a**) CTRL: 97.99 ± 1.81, n cells = 156; ATP: 143.80 ± 3.24, n cells = 158; IPA: 93.96 ± 2.75, n cells = 143; IPA + ATP: 81.00 ± 2.30, n cells = 160; (**c**) CTRL: 100.00 ± 1.09, n cells = 150; ATP: 116.33 ± 1.85, n cells = 150; IPA: 102.76 ± 1.48, n cells = 135; IPA + ATP: 93.22 ± 1.54, n cells = 137. (**b**,**d**) Representative fluorescent images (50×) of DAR4M-AM probe-labeled cells treated for 30 min (**b**) or 24 h (**d**). Images are presented in pseudocolor (LUT = fire) to better show the fluorescence intensity variations (range 11–120 (**b**), 11–89 (**d**)). IPA = indole-3-propionic acid, ATP = adenosine triphosphate.

To investigate the involvement of IPA in the control of vascular tone, we focused on its role in the modulation of endothelial NO production. For this purpose, experiments with the NO probe DAR4M-AM in fluorescence microscopy were performed. In these experiments, to simulate the physiological release of NO, we treated endothelial cells with the purinergic agonist adenosine triphosphate (ATP), proven to act as an autocrine/paracrine mediator of the flow-induced NO release [32]. In these experiments, we set four experimental conditions: BAE-1 cells in control conditions (CTRL); cells treated with 100 µM of ATP for 5 min (ATP); cells treated with 1 µM of IPA for 30 min (IPA, Figure 4a,b) and 24 h (IPA, Figure 4c,d); and cells treated for 30 min (Figure 4a,b) or 24 h (Figure 4c,d) with IPA plus ATP in the last 5 min (IPA + ATP). As shown in Figure 4, increased fluorescence intensity was observed only after ATP stimulation (positive control), while IPA alone did not induce an increase in NO levels, which remained the same as in the control; on the contrary, when BAE-1 cells were stimulated with both IPA and ATP, ATP failed to induce NO release. These results demonstrated that IPA does not alter basal NO production, but it is able to significantly reduce NO release in BAE-1 cells stimulated with ATP.

### 2.4. IPA Affects Endothelial Nitric Oxide Synthase (eNOS) Phosphorylation

**Figure 5:**
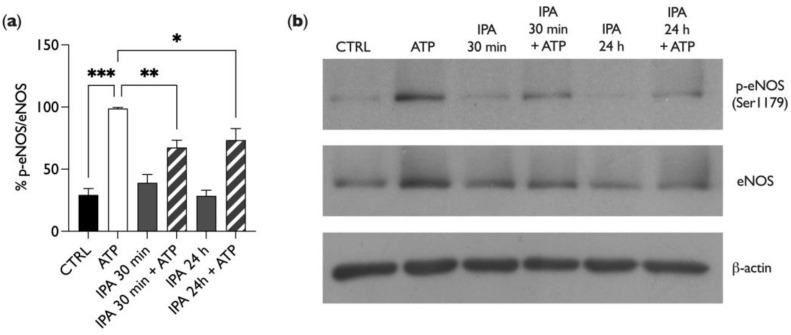
IPA regulates eNOS phosphorylation in BAE-1 cells. (**a**) Bar graph summarizing the p-eNOS/eNOS ratio normalized toward positive control (ATP) after treatment with 100 µM of ATP (5 min) and 1 µM of IPA (30 min and 24 h). Data in percentages are represented as mean ± SEM, * *p* < 0.05, ** *p* < 0.01, *** *p* < 0.001. Values of the n = 4 independent experiments were as follows: CTRL: 29.36 ± 5.15; ATP: 98.85 ± 0.86; IPA 30 min: 39.06 ± 6.64; IPA 30 min + ATP: 67.45 ± 5.88; IPA 24 h: 28.57 ± 4.55; IPA 24 h + ATP: 73.41 ± 4.55. (**b**) Representative Western blot experiment showing the effect of ATP and IPA on eNOS phosphorylation. Comparison of β-actin intensity ensured equal protein loading. IPA = indole-3-propionic acid, ATP = adenosine triphosphate.

To elucidate the mechanism involved in the IPA-dependent inhibition of NO release in BAE-1 cells stimulated by ATP, we performed a Western blot analysis of the Ser1179-phosphorylation level of endothelial nitric oxide synthase. It is indeed established that extracellular ATP binds to endothelial P2Y2 receptors, starting an intracellular cascade leading to Ser1179 eNOS phosphorylation (and consequent activation) [32]. For these experiments, the experimental conditions were the same as those described for Figure 4. As represented in Figure 5, ATP enhanced nitric oxide synthase phosphorylation, as expected. Interestingly, IPA treatment for both times did not affect eNOS phosphorylation, for which the levels remained the same as in the control (untreated cells). However, in the presence of IPA, ATP failed to increase the eNOS phosphorylation level, thus confirming the previous result on NO release. These data suggest that IPA reduces the purinergic-dependent NO release by lowering the Ser1179 phosphorylation level of eNOS.

## 3. Discussion

The present work studies the effect of indole-3-propionic acid (IPA), a tryptophan (Trp)-derived compound, on endothelial function. In particular, this research focuses on two main goals: first, to evaluate if IPA can induce cell toxicity when used in different concentration ranges on cultured bovine aortic endothelial cells (BAE-1), and, second, to monitor its role in modulating endothelial function parameters such as oxidative stress, nitric oxide (NO) release, and endothelial nitric oxide synthase (eNOS) activation through its phosphorylation.

Our interest moved from the widely accepted “gut–heart axis” hypothesis, which assumes a close relationship between gut microbiota-derived metabolites and cardiovascular health [33]. Indeed, different metabolites from the gut microbiota have already been related to cardiovascular disease (CVD); among all, trimethylamine N-oxide (TMAO) shows possible involvement in endothelial dysfunction [9,11,34], and short-chain fatty acids (SCFAs) seem to be involved in the improvement of atherosclerotic burden [35].

Trp is an essential amino acid for animal cells, so, to supply cellular needs, it has to be introduced through exogenous sources, such as diet. Part of the introduced Trp can be metabolized by entering one of the major pathways, namely serotonin, kynurenine, and indole pathways [12]. IPA is synthesized from the indoles pathway, primarily by gut bacteria from Clostridiaceae and Peptostreptococcaceae families [26,36], and it has been highlighted as a ligand for the nuclear receptors aryl hydrocarbon receptor (AhR) and pregnane-X receptor (PXR) [12,20,21], thus affecting protein expression. Like other secondary metabolites from the gut microbiota, IPA figures as a possible candidate able to influence CVD development [13,26]. The lack of consistent works pointing out its role in vascular health moved our interest to clarify if IPA could be considered a marker of endothelial dysfunction.

BAE-1 cells were treated with different concentrations of the compound, ranging from 10 nM to 5 mM, for 4, 24, and 48 h to assess if any endothelial toxic effect of IPA could be detected. As Figure 1 shows, only the over-physiological concentration of 5 mM after 48 h of treatment induced a significant reduction in cell viability. These results agree with those of Konopelski et al., who demonstrated that only a high IPA concentration had a cytotoxic effect on cultured human cardiomyocytes even at 24 h of treatment [24]. Moreover, Gesper and collaborators tested IPA on HL-1 cardiomyocytes, and none of the concentrations tested were toxic after 24 h of treatment [25], while Du and coworkers showed a significant increase in C2C12 cell viability after 24 h of treatment with IPA 0.1 mM [37]. 

According to the literature records [24,25,38], and to our first results that underline no cytotoxic effect of IPA, we decided to assess oxidative stress status by monitoring reactive oxygen species (ROS) production in a basal treatment with IPA and in combination with menadione (MEN), which has already been used to mediate oxidative stress-induced endothelial dysfunction by our and other groups [9,39,40,41].

Our results show no pro-oxidant or antioxidant effects of IPA both after 30 min and 24 h of treatment (Figure 2 and Figure 3); pre-treatment with the molecule was not able to induce ROS levels’ increase in the basal condition, and the simultaneous treatment with MEN did not change the effect of the stressor.

The same results were obtained by Gesper and collaborators, who did not observe ROS and hydroxyl radicals variations in HL-1 cells treated with IPA for 30 min and 24 h both in the basal condition and in the presence of a stressor [25]. Furthermore, only IPA concentrations higher than 2.5 mM were able to balance oxidative stress induced by iron in the skin [42], testes [43] homogenates, and hepatic microsomal membranes [44]. In opposition to these results, Sári and collaborators evinced increased oxidative/nitrosative stress through the upregulation of inducible nitric oxide synthase (iNOS), downregulation of nuclear factor erythroid 2-related factor 2 (NRF2), and increased mitochondrial oxidative stress in breast cancer cells treated with IPA [45]. These different results are probably due to the different cell models and techniques used to detect oxidative stress.

These first results underline that IPA is not involved in oxidative stress-induced endothelial dysfunction, thus confirming that there are no deleterious effects of the metabolite regarding cell viability.

Our study then moved towards considering the direct role of IPA in influencing nitric oxide (NO) release, the primary actor in the modulation of vascular tone [46]. As Jiang and collaborators comprehensively reviewed, human plasma concentrations of IPA settle between 50 nM and 1 µM in healthy subjects [36], so we decided to perform further experiments using only the concentration of 1 µM, which, in our experiments, was not toxic for BAE-1 cells.

One of the main physiological triggers of endothelial nitric oxide release is the purinergic pathway, with ATP among the most relevant modulators. After ATP binds the P2Y2 receptor on endothelial cells, a subsequent intracellular Ca^2+^ increase mediates the activation of eNOS through its phosphorylation at Ser1179 and the release of NO, directly involved in vascular smooth muscle cell relaxation [47]. Our first results monitoring NO in live cell imaging showed that IPA exerted a significant reduction in the ATP-induced gasotransmitter release both after 30 min and 24 h of incubation (Figure 4). Moreover, our data from Western blot experiments showed that IPA, both at 30 min and 24 h, counteracted the ATP-induced eNOS phosphorylation at Ser 1179 (Figure 5).

These results integrate that of Pulakazhi Venu and collaborators, who demonstrated a reduction in the relaxation of aortic tissue simultaneously treated with acetylcholine (Ach) and IPA [48]. In this study, the authors showed a PXR-dependent vascular effect of IPA, mainly mediated by the reduced expression of eNOS. In this context, the novelty of our data is to highlight an acute role of IPA, as both nitric oxide release and eNOS phosphorylation at Ser1179, that were reduced after 30 min of IPA stimulation, thus suggesting the involvement of a cytosolic-activated pathway, affecting eNOS activity, besides the PXR-dependent pathway, affecting eNOS expression. Indeed, most of the relevant differences between the two works can be traced back to a reduction in the expression of eNOS, as identified in Pulakazhi Venu’s paper, and there were no significant variations in eNOS expression in our results (Figure 5b). One of the possible reasons for this discrepancy can be explained by the diversity of the models used: experiments conducted on cell cultures involve inevitable differences from in vivo models. Moreover, our results were addressed to a functional evaluation of the effect of IPA on eNOS phosphorylation, an aspect that complements the data already presented in other works. Konopelski and collaborators showed an increased contractility of endothelium-deprived mesenteric resistance arteries treated with phenylephrine and IPA [24], and they supposed a direct constrictor effect of IPA on vascular smooth muscle rather than an endothelial-mediated effect. The discrepancies from our data could be ascribed to the different experimental protocols employed, as, in our cellular model, IPA negatively affected ATP-stimulated eNOS, while in Konopelski et al.’s study, IPA did not affect intact isolated mesenteric arteries precontracted with phenylephrine, thus probably figuring low-grade eNOS activation. 

In summary, the present paper shows for the first time the possible role of acute IPA exposure in the inhibition of ATP-stimulated eNOS phosphorylation at Ser1179, highlighting a potential negative impact of the molecule on the physiological control of vascular tone.

Inevitably, this study presents some limitations, such as, among others, the lack of a more detailed investigation of the mechanisms involved in IPA-mediated response, like other regulatory phosphorylations of eNOS, such as the inhibitory one at Thr495. Future in vivo investigations of the effect of IPA may be considered to corroborate the data presented here.

Moreover, this work presents some innovative aspects regarding the possible involvement of IPA in endothelial dysfunction; indeed, it clarifies that the effect of IPA is not mediated by oxidative stress and ROS, but rather it seems to directly influence NO release in endothelial cells. Furthermore, it is now widely accepted that IPA is a ligand for the PXR, and it could modulate nuclear transcription through this interaction. Besides the IPA-PXR-dependent modulation of vascular contractility [48], Du and coworkers showed the inhibition of NF-kB signaling and a reduction in proinflammatory cytokines secretion when IPA binds to PXR [37]. Our evidence of an acute effect of IPA on endothelial cells implies targets other than PXR, and future studies could investigate if IPA could affect one or more of the actors of the intracellular pathway managing NO release, such as calcium homeostasis, caveolar trafficking, protein kinase B (Akt), and others. In conclusion, this work enriches the pool of microbiota-derived compounds that affect cardiovascular health, in particular those involved in the development of endothelial dysfunction. IPA is not toxic for endothelial cells, and it does not induce a rise in ROS, but it does directly influence NO release, thus showing its possible role as a modulator of vascular tone. Deepening the effects of IPA could enrich knowledge of the interactions among diet, microbiota, and CVD. The discovery of a direct role of the molecule in the modulation of vascular function represents a fundamental starting point that physicians can consider suggesting to patients’ lifestyle interventions to be able to support pharmacological therapy or prevent pathological onset.

## 4. Materials and Methods

### 4.1. Materials

Unless otherwise specified, materials were obtained from Sigma Aldrich (Merck Group, Darmstadt, Germany). Plastics and reagents for cell cultures were obtained from Euroclone (Euroclone, Pero, Italy). 

Cell viability was assessed by the CellTiter 96^®^AQueous One Solution Cell Proliferation Assay (Promega Corporation, Madison, WI, USA) based on the use of the tetrazolium compound [3-(4,5-dimethylthiazol-2-yl)-5-(3-carboxymethoxyphenyl)-2-(4-sulfophenyl)-2H-tetrazolium, inner salt (MTS)]. The cell-permeant dye CellROX^®^ Green Reagent for oxidative stress detection was from ThermoFisher Scientific (ThermoFisher Scientific, Waltham, MA, USA).

Antibodies for immunoblotting experiments were purchased from the following suppliers: monoclonal anti-eNOS (code 610296) from BD (BD Biosciences, Franklin Lakes, NJ, USA), polyclonal anti-p-eNOS (code 36-9100) from ThermoFisher Scientific, and monoclonal anti-β-actin (code A5316) from Sigma Aldrich. Horseradish peroxidase-conjugated secondary antibodies (anti-mouse code 31430, anti-rabbit code SA00001-2) were both provided by ThermoFisher Scientific.

### 4.2. Cell Culture

Bovine aortic endothelial cells (BAE-1) were obtained from the European Collection of Authenticated Cell Cultures (ECACC, Salisbury, UK, No: 88031149). Cells were maintained in DMEM supplemented with 10% heat-inactivated fetal calf serum (FCS), 50 μg/mL of gentamicin, and 2 mM of glutamine in a humidified atmosphere of 5% CO_2_ in air. Cells were used at passages 2–6.

For fluorescence experiments on live cells, BAE-1 were plated in DMEM + 10% FCS on uncoated glass-bottom dishes, 35 mm diameter (Ibidi, Martinsried, Germany), at a density of 10,000 cells/cm^2^; after 48 h, cells were loaded with the appropriate probe and stimulated as planned.

For assays that require optical reading in a microplate reader (FilterMax F5TM Multi-Mode, Molecular Devices, Sunnyvale, CA, USA), BAE-1 cells were seeded in 96-well plates in DMEM + 10% FCS at a density of 10,000 cell/cm^2^ (3300 cells/well), maintained for 48 h in the cell incubator, and then treated as planned for the experiments.

### 4.3. Cell Viability Assay

As previously described [9], BAE-1 cells, grown in 96-well plates in DMEM + 10% FCS, were treated with DMEM + 10% FCS, either alone (control condition) or supplemented with IPA at different concentrations (10 nM, 100 nM, 1 µM, 10 µM, 100 µM, 1 mM, 5 mM) for three different treatment times (4, 24, or 48 h). Then, the effect on cell viability was estimated by staining with MTS, following the manufacturer’s protocol (10 µL of MTS added to 50 µL of medium in each well; six wells for each condition). MTS was bioreduced by metabolically active cells into a soluble and colored formazan product, and its absorbance, directly proportional to the number of viable cells, was read at 450 nm using the FilterMax F5TM microplate reader. Data were expressed as percentages of Abs referred to the control condition; percentage values of the three experiments were then analyzed to calculate mean ± SEM.

### 4.4. Reactive Oxygen Species (ROS): Microplate Reader

As previously described [49], BAE-1 cells, grown in black 96-well microplates with a clear bottom (Greiner Bio-One, Kremsmünster, Austria), were treated with DMEM + 10% FCS, either alone (control condition) or supplemented with IPA (100 nM, 1 µM, 100 µM) for 30 min or 24 h; treatment occurred with 20 µM of menadione (MEN) for 1 h to induce ROS production, which was then used as a positive control, while the addition of IPA and MEN simultaneously allowed for the recognition of IPA’s antioxidant effects. During the last 30 min of the stimulation, cells were loaded in the dark with 5 µM of CellROX^®^ Green Reagent, a probe that exhibits bright green fluorescence upon oxidation by ROS. Cells were washed twice with PBS, and fluorescence was acquired with the FilterMax F5TM microplate reader at excitation/emission of 485/535 nm. Data were expressed as percentages of fluorescence referring to the control condition; the percentage values of the three experiments were then analyzed to calculate mean ± SEM.

### 4.5. Reactive Oxygen Species (ROS): Fluorescence Microscopy

As previously described [49], BAE-1 cells, grown on glass-bottom dishes, were treated for 1 h with 20 µM of MEN to induce ROS production (positive control) or with 1 µM of IPA for 30 min or 24 h, alone or in combination with MEN, in DMEM + 10% FCS; cells were loaded with 5 µM of CellROX^®^ Green probe for the last 30 min in the dark. Cells were then washed twice with PBS, and fluorescence at 488 nm was acquired with a fluorescence inverted microscope (Olympus IX70, Olympus America Inc., Melville, NY, USA) with a 50× Uplan FI oil-immersion objective. Fluorescence intensity was evaluated in n = 15 random fields through the definition of the Regions of Interest (ROIs) using the software ImageJ (Rasband, W. S., ImageJ, U. S. National Institutes of Health, Bethesda, MD, USA; https://imagej.nih.gov/ij/. Version: 2.9.0/1.53t, 2010-2024 (accessed on 14 September 2022.), and the mean value of the resulting fluorescence for each condition was expressed as a percentage compared to the control of three independent experiments ± SEM.

### 4.6. NO Release

As previously described [49], BAE-1 cells, grown on glass-bottom dishes, were loaded with 5 µM of DAR4M-AM probe (Calbiochem^®^) for 30 min in the dark. Next, cells were washed twice with PBS and treated in PBS at 37 °C in the dark for 5 min with 100 µM of ATP as a positive control or 30 min with 1 µM of IPA alone or with ATP for another 5 min. For the set of experiments where IPA stimulation continued for 24 h, the DAR4M-AM probe was added for the last 30 min, followed by a further 5 min of ATP for co-treatments. Cells were washed twice with PBS, and fluorescence at 568 nm was acquired with the Olympus IX70 fluorescence inverted microscope at 50× magnification. Fluorescence intensity was evaluated in n = 15 random fields through the definition of the ROIs using ImageJ, and the mean fluorescence value for each treatment was expressed as a percentage compared to the control condition of the three independent experiments ± SEM.

### 4.7. Immunoblotting

As previously described [9,49], BAE-1 cells were seeded on plastic dishes, 20 cm^2^ of growth area, at a density of 10,000 cells/cm^2^, in 10% FCS DMEM, and grown in the cell incubator for 48 h. Cells were then treated with 100 μM of ATP for 5 min as a positive control or with 1 μM of IPA for 30 min or 24 h. Cell monolayers were lysed in 200 μL of RIPA lysis buffer (ThermoFisher Scientific) containing a phosphatase inhibitor cocktail (PhosSTOP, Roche, Mannheim, Germany) forced through a 1 mL syringe needle and centrifuged at 10,000 rpm for 5 min at 4 °C. Proteins (20 μg per lane) were resolved on 8% SDS-PAGE, transferred to as a polyvinylidene fluoride membrane (PVDF, ThermoFisher Scientific) in cold transfer buffer (25 mM Tris pH 8.3, 192 mM glycine, 0.1% SDS, 20% methanol) and blocked for 1 h at 37 °C in TBST (10 mM Tris–HCl, 0.1 M NaCl, 0.1% Tween 20, pH 7.5) plus 5% non-fat dry milk. Blots were incubated overnight at 4 °C with primary antibodies (1:500 monoclonal anti-eNOS; 1:250 polyclonal anti-p-eNOS; 1:2000 monoclonal anti-β-actin) in TBST containing 1% non-fat dry milk. Membranes were then washed three times with TBST and incubated for 1 h at room temperature with secondary antibodies (anti-mouse, 1:20,000, for monoclonal antibodies; anti-rabbit, 1:10,000, for p-eNOS) in TBST containing 1% non-fat dry milk, followed by a second set of three washes with TBST. Bands were visualized by chemiluminescence with Western Lightning Plus-ECL (Perkin Elmer, Waltham, MA, USA). Protein levels were determined using the software ImageJ; for each condition, the ratio of p-eNOS/eNOS was evaluated and then normalized against ATP as a positive control. The results of the n = 4 independent experiments were averaged and expressed as a percentage as mean ± SEM. The specific staining of secondary antibodies was checked; the comparison of the β-actin band intensity ensured equal protein loading.

### 4.8. Statistical Analysis

Data are presented as mean ± SEM. All data were analyzed with a one-way analysis of variance (ANOVA), followed by Bonferroni’s multiple comparisons for post hoc tests. Differences with *p* < 0.05 were considered statistically significant.

## Data Availability

Dataset available on request from the authors.
